# Patent data-driven analysis of literature associations with changing innovation trends

**DOI:** 10.3389/frma.2024.1432673

**Published:** 2024-08-01

**Authors:** Adrian Sven Geissler, Jan Gorodkin, Stefan Ernst Seemann

**Affiliations:** Center for non-coding RNA in Technology and Health, Department of Veterinary and Animal Sciences, University of Copenhagen, Frederiksberg, Denmark

**Keywords:** literature associations, biotechnology, big data, open data, patents

## Abstract

Patents are essential for transferring scientific discoveries to meaningful products that benefit societies. While the academic community focuses on the number of citations to rank scholarly works according to their “scientific merit,” the number of citations is unrelated to the relevance for patentable innovation. To explore associations between patents and scholarly works in publicly available patent data, we propose to utilize statistical methods that are commonly used in biology to determine gene-disease associations. We illustrate their usage on patents related to biotechnological trends of high relevance for food safety and ecology, namely the CRISPR-based gene editing technology (>60,000 patents) and cyanobacterial biotechnology (>33,000 patents). Innovation trends are found through their unexpected large changes of patent numbers in a time-series analysis. From the total set of scholarly works referenced by all investigated patents (~254,000 publications), we identified ~1,000 scholarly works that are statistical significantly over-represented in the references of patents from changing innovation trends that concern immunology, agricultural plant genomics, and biotechnological engineering methods. The detected associations are consistent with the technical requirements of the respective innovations. In summary, the presented data-driven analysis workflow can identify scholarly works that were required for changes in innovation trends, and, therefore, is of interest for researches that would like to evaluate the relevance of publications beyond the number of citations.

## 1 Introduction

The legal framework of patents and intellectual property laws is needed to provide an incentive for technological innovation and strengthen countries and their economies (Rockett, 2010). Yet, scientists have a surprisingly low patent literacy or are even in opposition to the core benefits patents have for societies (MacMillan, 2005; Peifer et al., 2021). Further, researches and administrators focus on the number of citations to determine the “scientific worth” of a scholarly work (Calver, 2022), while the number of citations is not linked to the patent relevance (Meyer, 2000). Therefore, there is a potential blind spot in identifying scholarly work that enabled technological innovation.

A patent is a *public disclosure* of a novel, non-obvious invention (claim), which an inventor is allowed to use *exclusively* for up to 20 years (Sheldon, 2009; Rockett, 2010). The exclusive usage time period starts either on the date of the patent application or at an earlier *priority date* on which a patent has been registered ahead of the full application. Additional patents can form a family of patents by broadening the scope or building on top of existing claims (Martinez, 2010). The written format of a patent application follows a fixed format, which includes a comparison to the state of prior-art and sufficient technical specification for an ordinary person skilled in the field to reproduce the invention (Sheldon, 2009). These parts of the application contain references to prior patents and scholarly works (journal articles, book chapters, and so on) that are of relevance for the invention.

Determining the degree of “relevance” that a scholarly work had on the creation of a patent is the matter of ongoing research (Verbeek et al., 2002; Li et al., 2014; Van Raan, 2017; Verbandt and Vadot, 2018). A concern in assessing the relevance of scholarly work is the practice of superfluous inclusion of scholarly work in the prior-art declaration due to regulatory policy (Verbeek et al., 2002; Verbandt and Vadot, 2018), such that the argument can be made that only the inventors know the true relevance of a scholarly work (Van Raan, 2017). Nevertheless, the scholarly works included in the prior-art declaration are crucial to the quality of the declaration for understanding and reproducing the invention (Verbeek et al., 2002; Li et al., 2014; Verbandt and Vadot, 2018). While existing methods that investigate in the linkage between science and technology focus on the relationship between patents, their inventors, and the authors of the works referenced in the prior-art declarations in order to determine the “relevant” works for individual patents (Li et al., 2014), *we propose to inspect the science-technology linkage on a larger scale for a set of patents that are connected to a specific technology*. In this study, we use the international patent classifications (IPCs) to collectively refer to all patents that are connected to a specific technology, *e.g*., gene regulation mechanisms in plants (Supplementary Table S1). Thereafter, we suggest to inspect the science-technology links with a statistical over-representation test to indicate which scholarly work is associated with a technology (instead of individual patents). This analysis approach is similar to methods commonly used in biomedical studies to identify disease pathways associated with genes ahead of expert follow-up inspection (Saelens et al., 2018).

To showcase the approach, we exclusively use publicly available data. Popular scientific databases, such as Google Scholar, the National Library of Health's PubMed platform, Scopus, or Clarivate's Web of Science, aggregate and index scholarly works and the references between them (Falagas et al., 2008). Similarly, databases can index patents based on the information publicly disclosed by patent offices. In contrast to Google's patent service which provides “*only”* a comprehensive index of patents,^1^ the Lens platform^2^ provides universal and equitable access to open innovation knowledge on a more complex information grid (Jefferson et al., 2021): The references between patents and scholarly works are captured, but also the collaborations between companies and universities, and the associations to individual inventors. Further, the Lens platform provides free access and interfaces to its platform for academic users, which allows for data science studies of patent innovation data.

Two biotechnological trends of high potential for sustainable food production and pharmaceutical development are based on patents that concern (i) the gene and genome modification technology CRISPR and (ii) photosynthesis capable cyanobacteria. The genome modifications with the CRISPR technology became possible after the discovery of the clustered regularly interspaced short palindromic repeats (CRISPR) system that bacteria use to defend themselves against pathogens (Cong et al., 2013). The CRISPR system uses short guide RNAs to target deoxyribonucleic acid (DNA) or ribonucleic acid (RNA) molecules that are complementary in sequence (Jinek et al., 2012). Similar to the CRISPR system in bacteria, artificial guide sequences can be designed to target specific genes in pursuit of a medical or biotechnological goal (Jefferson et al., 2021). One specific biotechnological application focus of CRISPR is metabolic engineering in cyanobacteria that are—due to the ability to perform photosynthesis, fixate nitrogen in agricultural soil, and produce nano particles—of relevance to medicine, carbon capture, food safety, and ecology (Behler et al., 2018).

For patents related to CRISPR or cyanobacteria, we performed time-series analyses to identify innovation trends through changes in the patent numbers according to the IPC. Afterward, we conducted an enrichment analysis to identify which scholarly works are significantly associated with the patents in a changing innovation trend. We identified multiple key publications that made possible the CRISPR technology and the biotechnological handling of RNA molecules and cyanobacteria. These results support our hypothesis that the presented workflow can identify scholarly works that have been significant in driving changes in innovation.

## 2 Materials and methods

### 2.1 Dataset of patent-cited literature

We used the data of patents and the corresponding referenced literature from the Lens platform (Jefferson et al., 2021). On the web platform, we created and downloaded two datasets for all patents that match the search terms “CRISPR” (downloaded July 6, 2023) and “cyanobacteria” (downloaded July 3, 2023).

The “CRISPR” dataset contains 60, 776 patents that reference 193, 517 scholarly works (journal articles, book chapters, etc). The “cyanobacteria” dataset contains 33, 489 patents and 84, 415 referenced scholarly works. The Lens platform provides 7, 288 international patent classifications (IPCs) for the “cyanobacteria” patents and 5, 118 classifications for the “CRISPR” patents. We matched these classifications to the World Intellectual Property Organization (WIPO) scheme (version 2023.01).

### 2.2 Identification of innovation trends through time-series analysis

For the downloaded patent-literature datasets, we counted the number of patents per priority year and IPC. We assume that the counts follow a negative binomial (NB) distribution, because the count mean μ and variance σ^2^ per IPC fit the characteristics of the NB distribution σ^2^ = μ+α·μ^2^ (linear model goodness of fit *R*^2^>0.92, Supplementary Figure S3B). The overall number of patents per year or per IPC does not correlate with the count per year and IPC (Supplementary Figure S2). However, the count per year and IPC does depend on the count in the preceding year (Pearson correlation 0.9, Supplementary Figure S3A).

The NB distribution is a mixture of a Poisson model with a Gamma distribution prior on the expected count parameter λ, that is the probability of observing a value for NB distributed random variable *X* (Cameron and Trivedi, 2013, chapter 8.2)


$$P_{NB}\left(X\right)=\int P_{Poisson}\left(X|\lambda \right)\cdot P_{Gamma}\left(\lambda |a,b\right)d\lambda$$

The parameters *a, b* of the Gamma distribution determine the variance and mean of the random variable. Following the composition of the distribution, we intuitively model the count of patents *X*_*ipc, year*_ in a year for an IPC as a time-series with a Markov-Chain such that the expected value is the number of patents in the preceding year with


$$P_{Poisson}\left(X_{ipc,year+1}|\lambda =X_{ipc,year}\right)$$

This Poisson probability of observing the change in number of patents by chance is computed for each IPC and year. The statistical significant changes in innovation trends are called for probabilities ≤ 10^−10^ and changes in number ≥100 (Supplementary Figure S3C).

### 2.3 Identification of scholarly work over-represented by innovation trends

For each dataset, we selected the top 10 IPCs with most significant trend change (by probability). We collected all patents and referenced scholarly works related to the selected IPCs. Afterward, we tested for each scholarly work if the number of references by patents is enriched for patents in any selected IPC compared to all patents in the dataset (one-sided Fisher-test, Supplementary Figure S4). We selected significant enrichment with false-discovery rate multiple-testing adjusted *P*-values ≤ 0.001 (the minimal probability of observing enrichment factors just by random chance). For computational reproducibility, we implemented the data processing and statistical tests in a Snakemake workflow (Koster and Rahmann, 2012), which is available together with both patent datasets under https://github.com/asgeissler/patent-data-explore.

## 3 Results and discussion

### 3.1 Most frequently patent-referenced scholarly works reflect science curriculum

**Figure 1:**
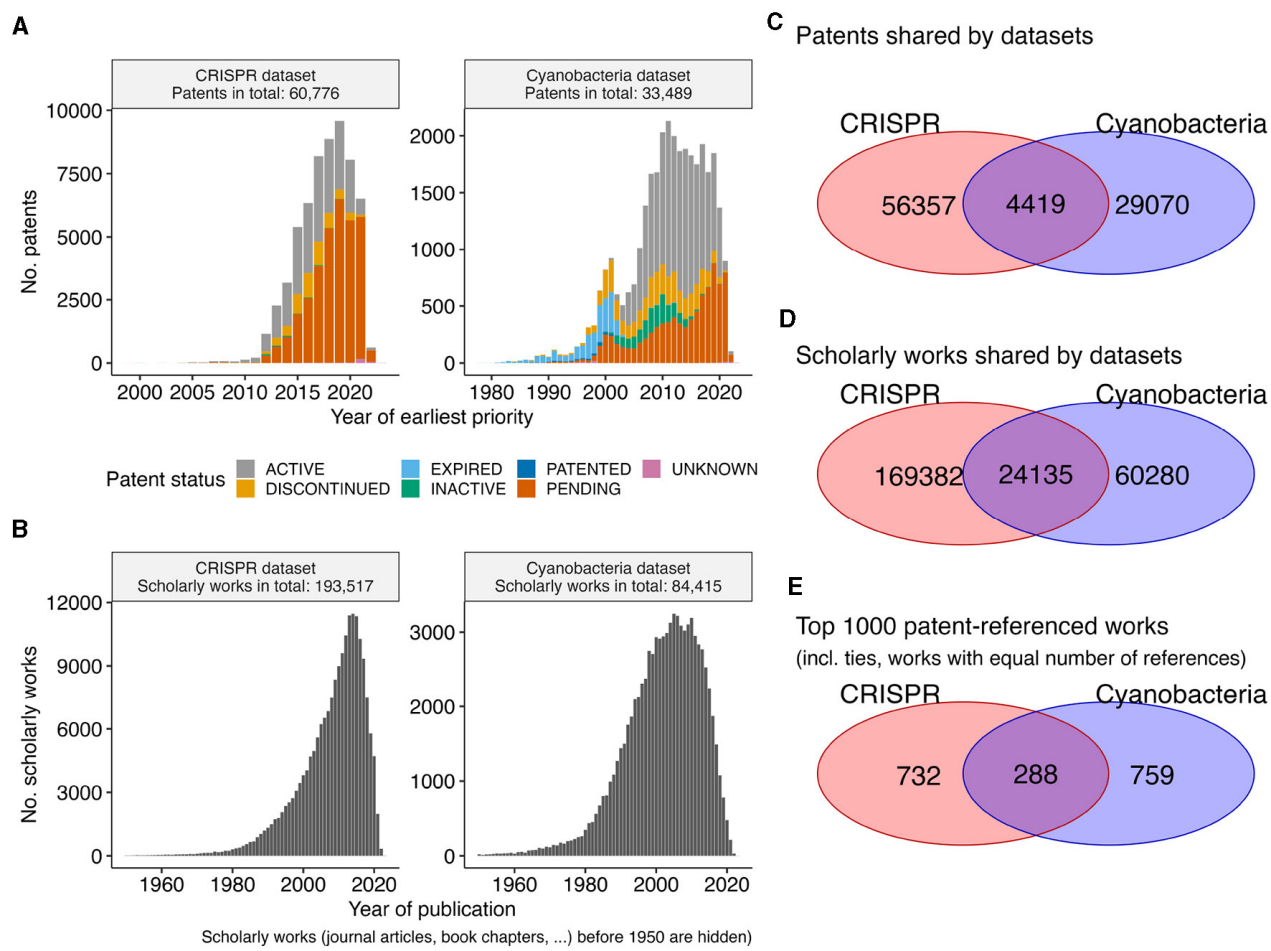
Overview patent datasets. **(A)** The bar plot shows the number of patents (*y*-axis) per year (*x*-axis) for the CRISPR and the Cyanobacteria dataset. Extra coloration indicates the current status of a patent (*e.g*., pending, …). The year refers to the priority year, if available, otherwise the year of the patent application. **(B)** The bar plot shows the number of scholarly works (*y*-axis) per published year (*x*-axis) to which the patents in each dataset refer. The Venn diagrams show the overlap between the two datasets for **(C)** patents, **(D)** referenced scholarly work, and **(E)** the top 1,000 referenced scholarly works.

We inspected patent data and their associated scholarly work for the two biotechnological datasets on CRISPR technologies and photosynthesis competent cyanobacteria. These two datasets were chosen according to the expertise of the authors, in order to evaluate any downstream literature associations. Consistent with the maximal 20 year exclusive usage time frame of patents, all patents with priority dates in the year 2000 or earlier have expired (Figures 1A, B). While the Cyanobacteria dataset contains patents since the year 1980, the bulk of its patents are from 2004/2005 or later. In contrast, the patents of the CRISPR dataset represent a more recent aspect of biotechnology, with the earliest patents having priority years around 2000. Nevertheless, the more recent CRISPR dataset has nearly twice as many patents than the Cyanobacteria dataset (60, 776 vs. 33, 489). Underlining the general importance of CRISPR technologies (Jefferson et al., 2021), both datasets share 4, 419 patents (Figure 1C) with shared references to 24, 135 scholarly works (Figure 1D). However, these overlaps in relation to the general dataset are minor (< 10% Jaccard similarity, that is the size of the intersection over the size of the union). Even when focusing on the top 1, 000 most referred scholarly works per datasets, the overlap is small (< 17% Jaccard similarity, Figure 1E). Therefore, we consider these two datasets as two separate datasets for exploration.

While the patents are referring to scholarly works published in “big journals,” such as Nature, Science, PNAS, NAR, *etc*, the bulk (>95%) of scholarly works were published in a diverse set of ~10,500 journals (Supplementary Figure S1A). Consistent with prior observations (Meyer, 2000), the number of citations a scholarly work has is not correlated with the number of referring patents (Pearson correlation 0.16, Supplementary Figure S1B). The selection of scholarly works with the most referring patents per dataset (Supplementary Figure S1C) revels the scientific foundational work, such as the publications of Jinek et al. (2012) and Cong et al. (2013) that first characterized the CRISPR system in bacteria and the system's application in genome engineering. Since CRISPR-based engineering requires knowledge about genomic sequences and their corresponding positions in genomes, it is to be expected that the publication of the Basic Local Alignment Search Tool (BLAST) by Altschul et al. (1990) is among the scholarly works with the most patents references, because the sequence search functionality of the BLAST tool is an essential component of any bioinformatics teaching curriculum (Welch et al., 2014). The core genetic curriculum includes the quantitative analysis of association between genetic markers and phenotypical trait (Miles and Wayne, 2008), which is consistently represented in the datasets by the references to Eshed and Zamir (1996) and Kraft et al. (2000). Similarly, patents in the Cyanobacteria frequently refer to the publications of Hallauer et al. (2015) and Wych (2015) that elucidate seed handling and engineering for improved agricultural yields. Overall, recovering these frequently referenced publications might be of interest for a reader new to the respective discipline, and these core curriculum publications are essential in the development of the underlying technologies. Newer development within the discipline, however, might be enabled by more specialized scholarly works.

### 3.2 Innovation trend changes are consistent with changes in technology and application

**Figure 2:**
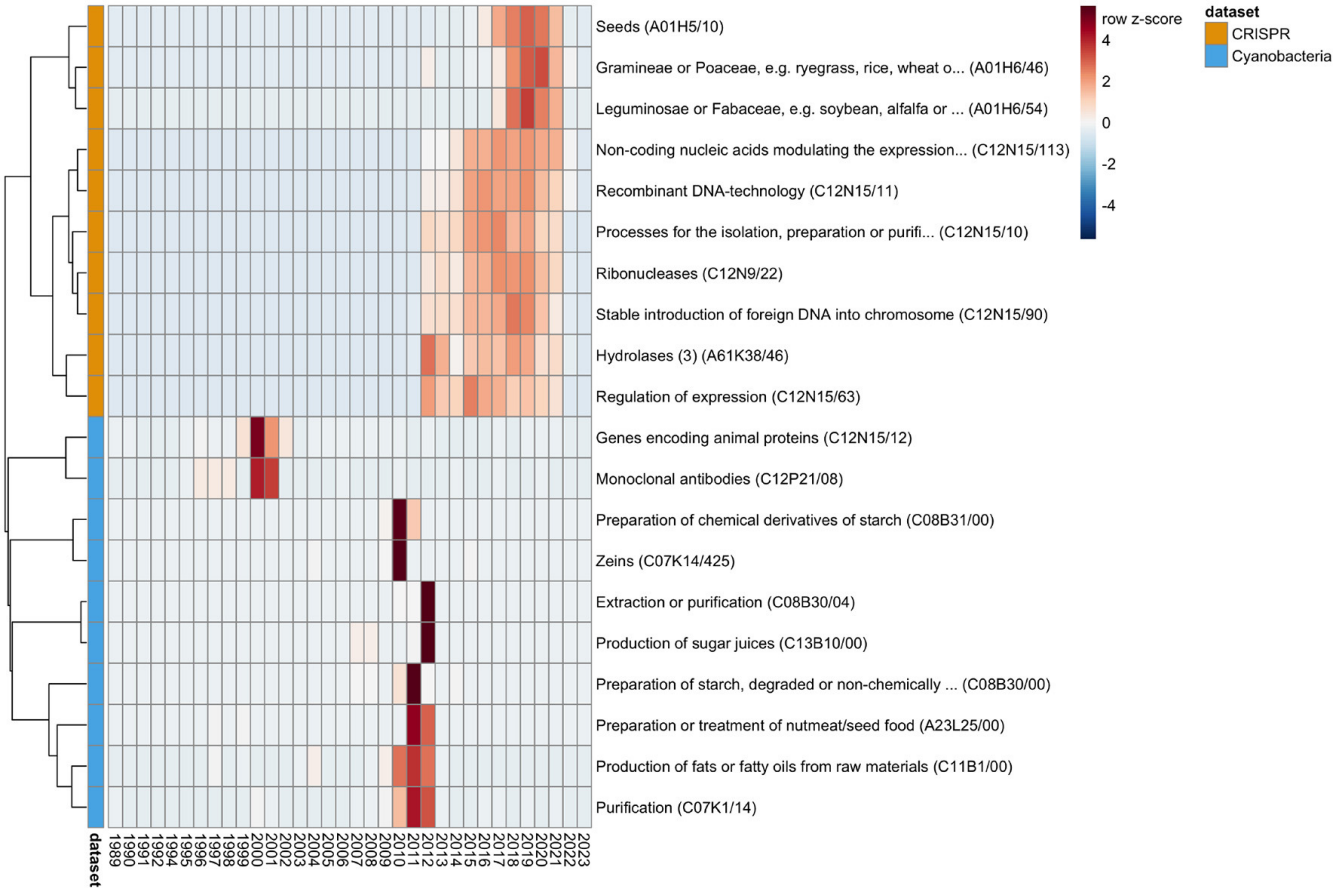
Trends in patent numbers. The time-series analysis identified trends of significant changes in patent numbers. The figure shows the top 10 IPCs for the CRISPR (orange) and Cyanobacteria (light blue) datasets. The heatmap shows per IPC (row) the *z*-scaled (thus the number of standard deviations from the average) patent numbers per year (column). The IPCs (rows) are ordered according to a hierarchical clustering (complete linkage of the Euclidean distances between the *z*-scaled values) in order to arrange IPCs with overall similar trends near each other. Red coloration indicates a number of patents above per IPC average, and blue indicates a below average patent number. Due to the *z*-scaling, the visual patterns allow for comparison of trend changes between IPCs, but not in absolute levels. Figure created with pheatmap (Kolde, 2019).

We analyzed the time-series data for the number of patents per IPC (Supplementary Figure S3), and identified statistically significant trend changes of greater or equal than 100 new patents during one year with a probability of by chance observation ≤ 10^−10^ (which is 34 × less likely than winning the grand price in the Powerball lottery^3^). For further investigation, we selected the top 10 IPCs per dataset by the minimal probability observed at any time-point (Figure 2). By visual inspection of the patent numbers over time for the selected IPCs we observed the following:

- The number of patents for monoclonal antibodies and expression of animal proteins increased rapidly in the 1990s, but has decreased since, which might be related to the scientific interest in that time period (Metcalf and Codd, 2003)
- Although algae have been part of the human nutritional plan since ancient times, cyanobacteria, and microalgae in general, got increased attention by high-tech industries to create novel food options with the beginning of the 21st century (Becker, 2013; Wells et al., 2017; Torres-Tiji et al., 2020). This context might explain the stark increase in patents for food production and agriculture related IPCs (*e.g*., production of sugar juices) in the years 2010–2012.
- Since the CRISPR-based gene editing technology has been developed only recently (>2010) (Jinek et al., 2012; Cong et al., 2013), the largest trend changes in the CRISPR dataset are related to technical aspects of developing the technology (*e.g*., for the regulation of expression or stable insertion of foreign DNA into genomes).
- The trend of using CRISPR technologies for the engineering of seeds in agriculture has only started in 2016/2017 (Zhang et al., 2020). However recent updates in regulatory legal frameworks have the potential of leading to substantial increase in research of development in agricultural biotechnology (Mehta, 2023; Vora et al., 2023).

Although the visual patterns in the patent numbers of the selected IPCs might seem to fall below average in 2022/2023, the decrease is consistent with the overall decrease in numbers for both datasets (Figure 1). We suggest that this decrease in number is not a reflection of the actual decrease in patent applications in recent years, rather the respective applications are not yet publically available, and thus not contained in the downloaded datasets. Consistently, the average number of years between the date of application and date of publication by the patent office is 2.9 ± 2.2 across both datasets.

The above listed items support that the time-series analysis is able to successfully identify changes in patent numbers that are consistent with changes in trends of scientific interest and technology innovations reported in the literature.

### 3.3 Scholarly works about specific innovations are over-represented by trend changes

**Figure 3:**
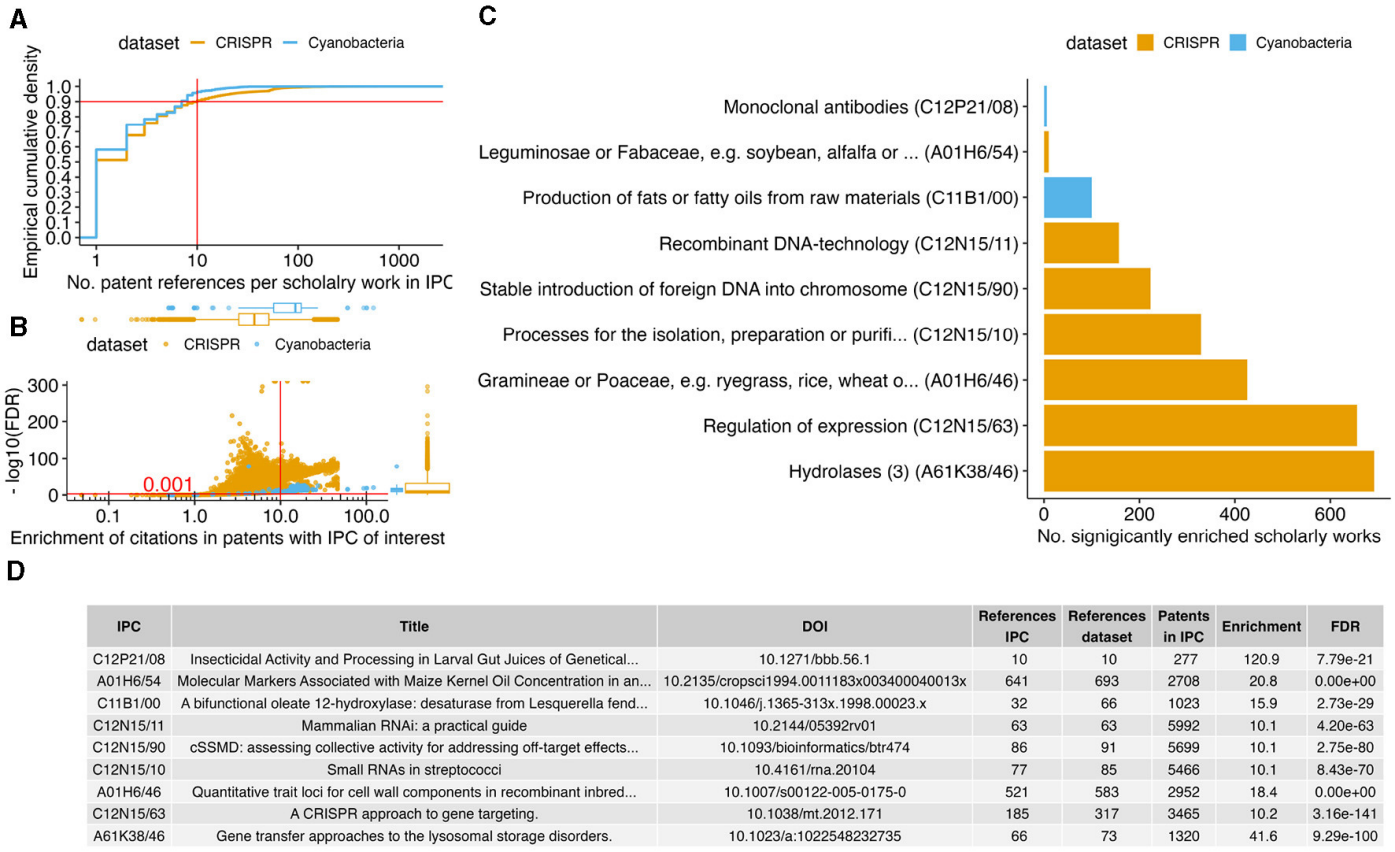
Innovation trend associated scholarly works. **(A)** For each IPC of interest (Figure 2), the sub-plot shows the distribution of the number by how many patents each scholarly work was referenced with the empirical cumulative density. The horizontal and vertical lines indicate the cutoff for the minimal number of referencing patents. For each scholarly work above this cutoff, we conducted an over-representation test of references per IPC. **(B)** The scatter plot shows the enrichment in observed over expected number of references (*x*-axis) against the log scaled FDR adjusted *P*-value (*y*-axis) We selected for at least 10-fold enrichment with FDR ≤ 0.001 (red lines). **(C)** The bar plot shows the number of scholarly works (*x*-axis) that are significantly enriched per IPC (*y*-axis). **(D)** The table shows per IPC the most significantly enriched scholarly works (ties in FDR were resolved by enrichment factor and most recent publication). The rows in the table are ordered according to the *y*-axis in the bar plot **(C)**.

After the identification of IPC-encoded biotechnological patent trends with statistical significant changes in patent numbers over time (Figure 2), we tested if the corresponding patents have an over-represented number of references to specific scholarly works in comparison to the other patents of each dataset. This approach has the potential of finding scholarly works that are specific to an innovation trend. The methodology is similar to what is commonly used in molecular biology for assessing if a cluster of genes is associated with a disease pathway (Saelens et al., 2018). After filtering for scholarly works that were at least 10-fold enriched in the references in the top 2 × 10 IPCs (false discovery rate 0.1%, Figures 3A, B), the IPCs for patents that regulate expression or use hydrolase enzymes (breaking chemical bonds under usage of water) still had >600 enriched scholarly works. In total, a set of 1,078 scholarly works were enriched in 9 IPCs of the top 20 IPCs (Figure 3C); most (7) IPCs with enriched scholarly works originate from the CRISPR dataset. When focusing on the most significant scholarly work per IPC (Figure 3D), we observed consistency with the theme of the corresponding IPC (in order of publication year):

- Le Rhun and Charpentier (2012) review the regulatory RNAs and the CRISPR system of human pathogenic *Streptococcus* bacteria. The review includes a discussion on methods for extracting and detecting RNAs in the pathogen, consistently, this scholarly work was the most enriched paper on patents in the CRISPR dataset for processing, isolating, purifying RNAs.
- Carroll (2012) outlines the use of CRISPR to target and modulate expression of genes, and is enriched in the corresponding IPC.
- Zhang et al. (2011) characterized the RNA-based interference and targeting of gene expression (RNAi) with respect to unintended “off-target effects” in the genome. The consideration for off-targets is a core requirement for successful CRISPR-based genome editing (Anthon et al., 2022). Consistently, the paper is over-represented in references by patents from the CRISPR dataset aiming at inserting foreign DNA into genomes.
- Krakowsky et al. (2006) quantitatively analyzed the genetic association to the cell wall components of maize plants, and is enriched in the plant family IPC (botanical family of Gramineae and Poaceae).
- RNAi requires the insertion of genetic material into a chromosome; the handbook on that method by Sandy et al. (2005) was consistently enriched in patents involved in recombinant DNA-technology.
- Barranger et al. (1999) characterized the model system to develop therapies for lysosomal storage disorders; the lysosome is an organelle that processes organic compounds with hydrolase enzymes.
- Broun et al. (1998) were first in exploring the genetic factors behind the fat content of plant seeds. Consistently, their paper was enriched in patents on the topic of fat/oil production.
- The methods used by Goldman et al. (1994) to cross maize kernel for an increased oil yield may also be applicable for other plants, such as soybeans. Consistently, the paper by Goldman et al. (1994) is enriched in references by patents involving soybeans.

All of these publications are consistent with the IPC they were significantly often cited in. However, the statistical significant association in enrichment between the publication by Nakamura et al. (1992) and patents on monoclonal antibodies is not immediately apparent. The publication has < 30 citations (both in the Lens database and in the Google Scholar index), such that current metrics on academic success might not rank this publication highly (Calver, 2022). However, the work by Nakamura et al. (1992) might have been essential (due to citation) in forming the two highly cited immunology related (and thus the potential antibody patents enrichment) papers of Uzé et al. (1995) (243 literature citations and 24 patent references in Lens) and Rukmini (2000) (60 literature citations and 17 patent references). Our analysis suggest that the work of Nakamura et al. (1992) had been more relevant for patented innovation development than its recognition in scholarly citations (Meyer, 2000).

Related to the legal framework of patents, this study's analysis methodology cannot identify if the change in patent numbers had been due to external changes in the societal or judicial context. Further, the scholarly works that are statistically associated with an IPC trend might become of interest in a different innovation trend with technological progress by “lucky coincidence” (serendipity), similarly to how the drug sildenafil (Viagra) that was initially designed for anti-angina medication can be used in the treatment of erectile dysfunction and cancer (Goldstein et al., 1998; Meyer et al., 2011; Prasad et al., 2016).

Concerning the editing of genes with CRISPR, particularly in the genome of plants: The European Parliament is currently (state 2023/2024) in the process of passing new laws that will abolish patents for genetically modified organism if the modified organism closely resembles a genome that could have originated from a conventional breeding procedure (Vora et al., 2023). While the laws are not yet passed, the new regulatory framework has the potential to boost new genomic techniques that could lead to a boost of biotechnological and agricultural innovation in the European Union (Mehta, 2023). Consequentially, future work could investigate the impact of these new regulations on the patent and literature data in the coming years.

## 4 Conclusion

In this study, we applied methods commonly used in associating genes to disease pathway in molecular biology to publicly available patent information (Saelens et al., 2018). Specifically, we aimed at detecting scholarly works that are statistically significant in association with a subset of patents that represent an area of innovation with substantial trend changes in patent numbers over time. In two datasets of patents, we demonstrated that the overall number of patent references can identify scholarly works that formed the basis for a technology or can be considered as part of a teaching curriculum in the field, for example the methodologies of the CRISPR technology published by Jinek et al. (2012) and Cong et al. (2013). However, only the over-representation test found the associations with specific innovation, such as the scholarly work on off-target considerations for CRISPR that is required and thus enabled the stable insertion of foreign DNA into genomes (Zhang et al., 2011; Anthon et al., 2022). While the over-representation analysis points out these associations of interested, additional expert knowledge and literature research was needed to sanitize the plausibility of each association that was found by the statistical analysis. This “limitation” is common to the application of an over-representation analyses in the biomedical field: The statistical analysis provides researchers “only” with a data-driven list of genes that might be associated with a disease, but additional experiments are needed to validate if a gene is causal to disease (and thus a candidate for drug development). The advantage of the over-representation analysis is that it provides a starting point for subsequent inspection, which greatly benefits the biomedical research, because the number of genes is too large for “randomly” probing genes as potential drug targets (Wishart, 2006). Similarly, the application of the over-representation analysis on patent data provides a starting point for identifying scholarly work that could be of interest to read in the context of a technological innovation.

Our approach can help to evaluate the significance of scholarly publications beyond the number of citations in other scholarly works (Calver, 2022), but also beyond references in individual patents, which due to regulatory policies inflates the references to foundational work in a field (Verbeek et al., 2002). Instead, this methodology provides a data-driven approach to effectively rank literature according to their statistical significance of association to an entire class of patents, and to highlight specific scholarly work associated with a changing trend in technology. We consider our method to be of interest for researchers that would like to review literature in emerging disciplines or would like to identify key literature associated with a specific sub-field of an innovation.

## Data availability statement

Publicly available datasets were analyzed in this study. This data can be found at: https://github.com/asgeissler/patent-data-explore.

## Author contributions

AG: Conceptualization, Formal analysis, Investigation, Methodology, Writing – original draft, Writing – review & editing. JG: Funding acquisition, Supervision, Writing – review & editing. SS: Funding acquisition, Supervision, Writing – review & editing.

## Footnotes

^1^ https://patents.google.com/

^2^ https://www.lens.org/

^3^ https://www.powerball.com/powerball-prize-chart
