# Supplementary material for: Patent data-driven analysis of literature associations with changing innovation trends
Source: Front Res Metr Anal. 2024 Aug 1;9:1432673. doi: 10.3389/frma.2024.1432673 (PMC11324476; doi:10.3389/frma.2024.1432673)
Supplement: Supplementary file 1 [file Data_Sheet_1.PDF]

Supplementary Material

PATENT DATA-DRIVEN ANALYSIS OF LITERATURE ASSOCIATIONS WITH CHANGING INNOVATION TRENDS

Adrian Sven Geissler [1], Jan Gorodkin [1], and Stefan Ernst Seemann [1]

[1] Center for non-coding RNA in Technology and Health, Department of Veterinary and Animal Sciences, University of Copenhagen, 1870 Frederiksberg, Denmark

Table S1. *IPC classification scheme illustration.* The international patent classification (IPC) is a hierarchical system of sections, classes, levels, and groups with multiple, potentially nested, sub-groups. (A) The tables illustrate the classification of biotechnological patents using the sub-group for probabilistic gene networks. (B) The current IPC scheme contains up to 9-fold nested subgroups. The titles of subgroups start with dot symbols '•'. Further, the titles might require consulting the hierarchy for human interpretation. For example, the IPC "C12N 15/82" has the title of only "for plant cells". Without the scheme, it would not be clear that a patent with this classification introduces foreign genetic material into a plant cell to regulate gene expression.

A

Classification	Symbol	Title
Section	G	Physics
Class	G16	INFORMATION AND COMMUNICATION TECHNOLOGY (ICT)
Level	G16B	BIOINFORMATICS
Main-group	G16B 5/00	ICT specially adapted for modelling or simulations in systems biology, e.g. gene-regulatory networks, protein interaction networks or metabolic networks
Subgroup	G16B 5/20	• Probabilistic models

B

Classification	Symbol	Title
Section	C	CHEMISTRY; METALLURGY
Class	C12	BIOCHEMISTRY
Level	C12N	MICROORGANISMS OR ENZYMES
Main-group	C12N 15/00	Mutation or genetic engineering
Subgroup	C12N 15/09	• Recombinant DNA-technology
Sub-subgroup	C12N 15/63	• • Introduction of foreign genetic material using vectors; Vectors; Use of hosts therefor; Regulation of expression
Sub-sub-subgroup	C12N 15/79	• • • Vectors or expression systems specially adapted for eukaryotic hosts
Sub-sub-sub-subgroup	C12N 15/82	• • • • for plant cells

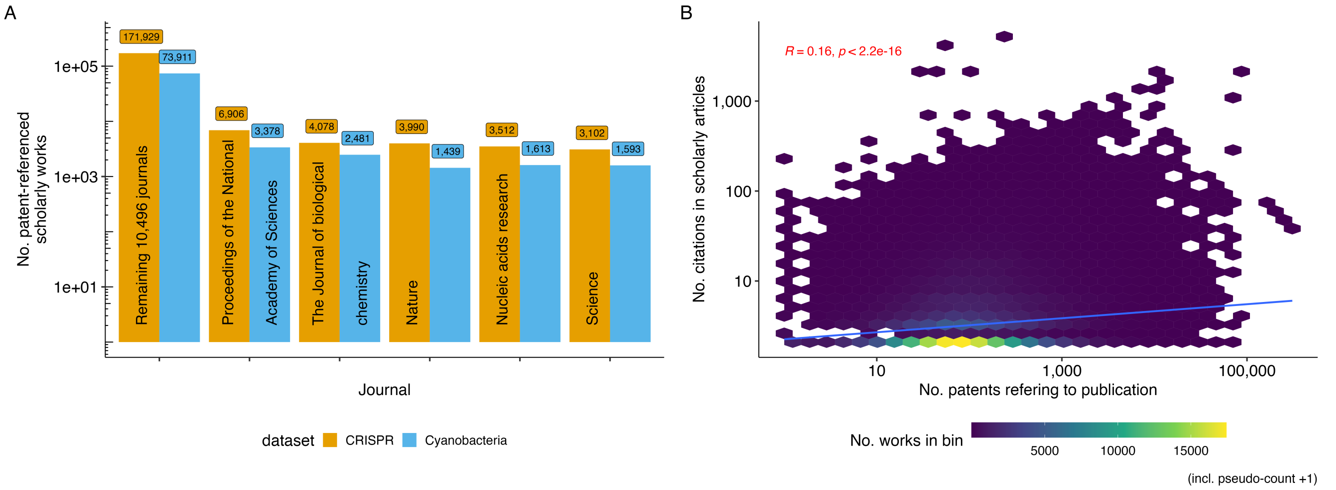

C

Dataset	References in dataset	References globally	Citations	Title	Authors	Journal/Publisher	Year	DOI
Cyanobacteria	3756	6,629	235	Less-than-additive epistatic interaction...	Yuval Eshed, et al.	Genetics	1996	10.1093/genetics/143.4.1807
Cyanobacteria	3756	6,621	52	Linkage disequilibrium and fingerprintin...	T. Kraft, et al.	Theoretical and Applied Genetics	2000	10.1007/s001220051486
CRISPR	2364	2,422	11,531	A programmable dual-RNA-guided DNA endon...	Martin Jinek, et al.	Science	2012	10.1126/science.1225829
Cyanobacteria	2102	2,756	91	Inbreeding Depression, Inbred and Hybrid...	M. R. Meghji, et al.	Crop Science	1984	10.2135/cropsci1984.0011183x002400030028x
Cyanobacteria	2101	2,753	37	Corn Breeding 1	Arnel R. Hallauer, et al.	Agronomy Monographs	2015	10.2134/agronmonogr18.3ed.c8
Cyanobacteria	2100	2,725	27	Corn and Corn Improvement - Production o...	Robert D. Wych	Agronomy Monographs	2015	10.2134/agronmonogr18.3ed.c9
CRISPR	1940	6,621	52	Linkage disequilibrium and fingerprintin...	T. Kraft, et al.	Theoretical and Applied Genetics	2000	10.1007/s001220051486
CRISPR	1929	6,629	235	Less-than-additive epistatic interaction...	Yuval Eshed, et al.	Genetics	1996	10.1093/genetics/143.4.1807
CRISPR	1800	1,825	11,916	Multiplex Genome Engineering Using CRISP...	Le Cong, et al.	Science	2013	10.1126/science.1231143
CRISPR	1512	15,019	80,273	Basic Local Alignment Search Tool	Stephen F. Altschul, et al.	Journal of molecular biology	1990	10.1016/s0022-2836(05)80360-2

Figure S1. *Patent dataset structure.* (A) The bar plot shows the number of scholarly works (y-axis) for the top 5 journals (x-axis). The remaining 10496 journals are shown as ”other”. The number of works is split per dataset, as indicated by coloration. (B) The hexagonal heatmap plots the number of patents that refer to each scholarly work (x-axis) versus the number of citations by other scholarly works (y-axis). The linear regression line is shown in blue, with the Pearson correlation and its significance stated in the red labels. The color gradient indicates the number of works per hexagon. (C) The table lists for the Cyanobacteria and the CRISPR dataset the top 5 scholarly works with most references.

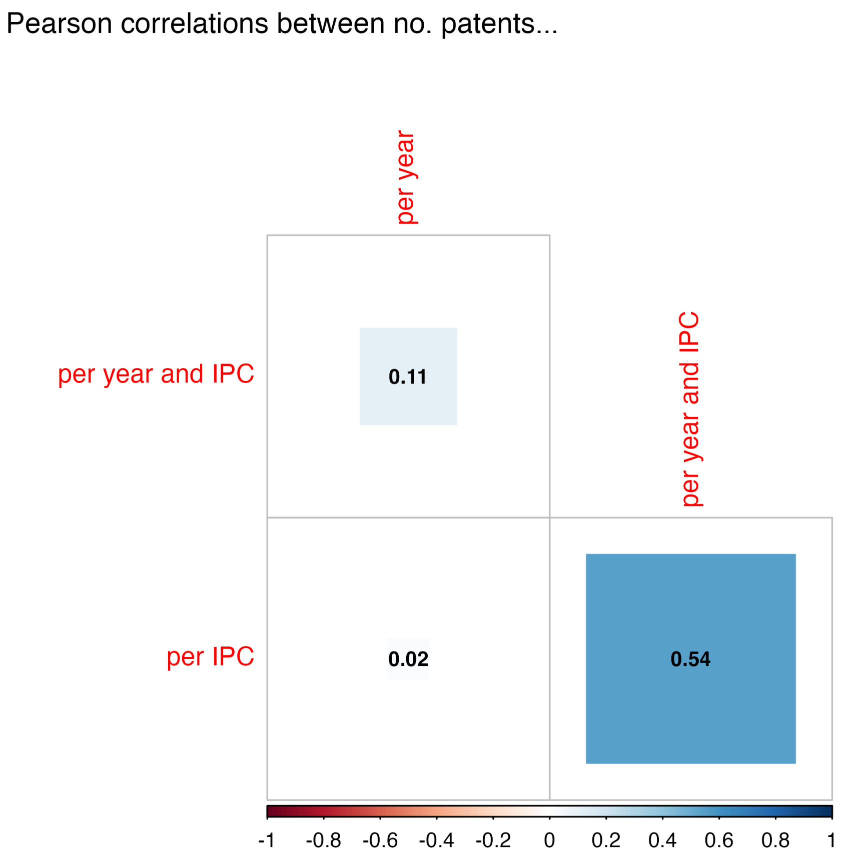

Figure S2. *Correlation of patent numbers.* The figure shows the Pearson correlations (black numbers) between the following statistics on the count matrix with the number of patents with a certain international patent classification (IPC, rows) and per year (columns): (i) The number of patents with an allocated IPC (sum of the numbers per row), (ii) the number of patents in a year (sum of patents per column but excluding double counting of patents with multiple IPCs), (iii) the patents per year and IPC (the individual matrix cells). For visual aid, colored boxes indicate the amount of correlation (red-blue gradient).

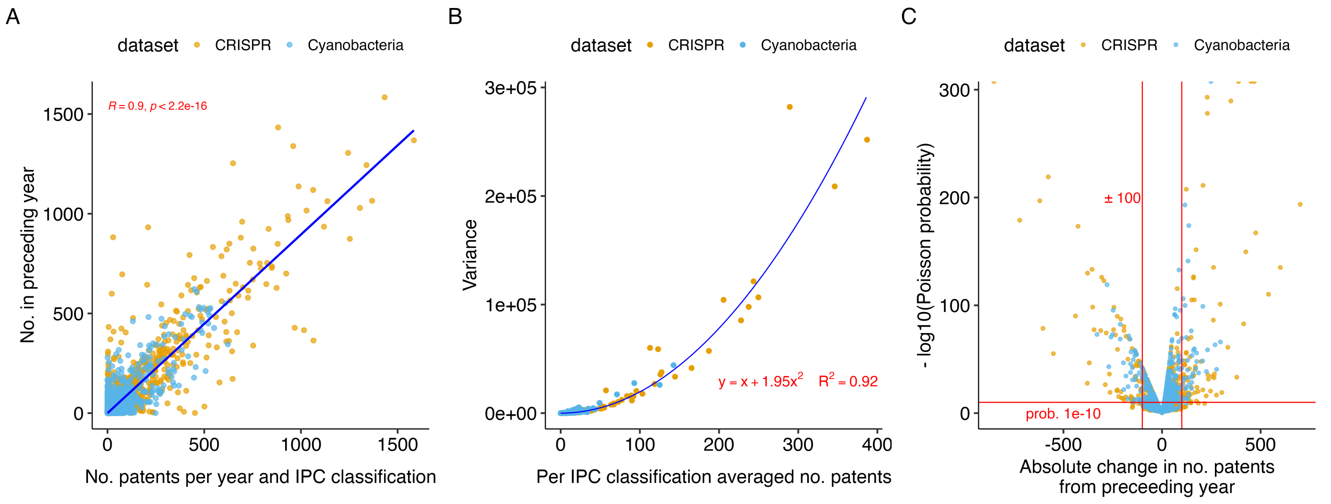

Figure S3. *Distribution characteristics.* (A) The scatter plot shows the number of patents per IPC and year (x-axis) against the number in the preceding year (y-axis). The linear regression (blue line) and Pearson correlations (red text) are as shown. (B) The scatter plot shows the mean number of patents (x-axis) versus the variance (y-axis). The red labels state the equation of a linear model for the mean-variance relationship according to the polynomial characteristics of a negative binomial distribution. The blue line illustrates the fitted models. (C) The volcano-like scatter plot shows the difference in number of patents from one year to the next (x-axis) against the negative log scaled Poison probability, which has the number of patents in the preceding year as expected number parameter (y-axis). The vertical and horizontal red lines indicate the cutoffs used in the study (absolute number change ≥ 100 and probability $\leq 10^{-10}$. In all sub-plots, the points are colored according to the corresponding dataset (orange and blue).

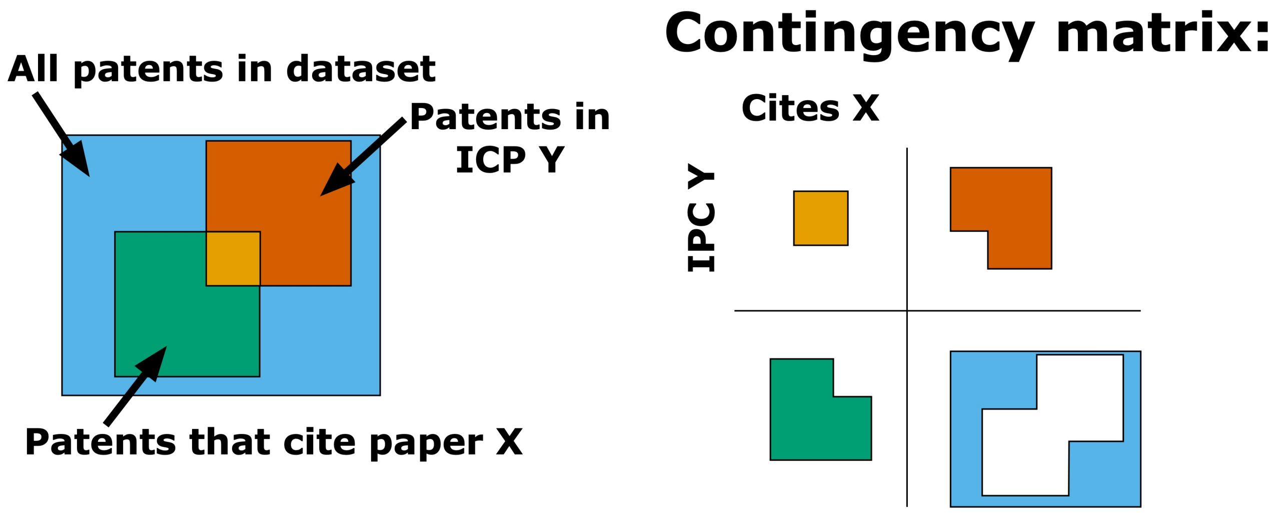

Figure S4. *Citation enrichment test.* In this study, we test for over-representation of citations for each paper X by patents in each IPC Y that had a significant change in the trend of patent numbers over time. The Fisher test compares the odds in numbers of patents citing X and being part of Y (orange) relative to the other remaining citing X (green) in comparison to the patents that in Y but do not cite X (red) relative to all remaining patents in the dataset (blue). The number for each of these cases fill the contingency matrix, that is the input to the one-sided fisher test.
